# Fatigability in spinal muscular atrophy: validity and reliability of endurance shuttle tests

**DOI:** 10.1186/s13023-020-1348-2

**Published:** 2020-03-23

**Authors:** Bart Bartels, Janke F. de Groot, Laura E. Habets, Camiel A. Wijngaarde, Wendy Vink, Marloes Stam, Fay-Lynn Asselman, Ruben P. A. van Eijk, W. Ludo van der Pol

**Affiliations:** 1 Wilhelmina Children’s Hospital, University Medical Center Utrecht, Child Development and Exercise Center, Lundlaan 6, Internal mailbox no. KB 02.056.0, Utrecht, 3508 AB The Netherlands; 2 Research Group Lifestyle and Health, HU University of Applied Health Sciences Utrecht, Utrecht, The Netherlands; 3 University Medical Center Utrecht, UMC Utrecht Brain Center, Utrecht, The Netherlands; 4 Rijndam Rehabilitation Center, Rotterdam, The Netherlands; 5 University Medical Center Utrecht, Biostatistics & Research Support, Julius Center for Health Sciences and Primary Care, Utrecht, The Netherlands and University Medical Center Utrecht, UMC Utrecht Brain Center, Utrecht, The Netherlands

**Keywords:** Spinal muscular atrophy, SMA, Fatigability, Endurance, Outcome measure, Muscle fatigue, ESNHPT, ESBBT, ESWT, ESTCS

## Abstract

**Background:**

To determine construct validity and test-retest reliability of Endurance Shuttle Tests as outcome measures for fatigability of remaining motor functions in children and adults with Spinal Muscular Atrophy (SMA) across the severity spectrum.

**Results:**

We assessed the Endurance Shuttle - Nine Hole Peg Test (ESNHPT), − Box and Block Test (ESBBT) and – Walk Test (ESWT) in 61 patients with SMA types 2–4, 25 healthy controls (HC) and 15 disease controls (DC). Convergent validity, discriminative validity and test-retest reliability were investigated. Additionally, we compiled the Endurance Shuttle Combined Score (ESTCS) by selecting the most relevant endurance test of each individual. 54, 70 and 73% of patients with SMA demonstrated increased fatigability on the ESNHPT, ESBBT and the ESWT. Endurance response in SMA was characterized by a decrease in muscle strength, an increase in muscle fatigue and an increase in motor adaptions, thereby confirming convergent validity. Patients with SMA showed increased drop-out rates and a shorter endurance time compared to HC and DC demonstrating good discriminative validity. Test-retest reliability was moderate to excellent (ICC’s ranging from .78 to .91) with a trend towards better performance on retest. The ESTCS increased sample size and drop-out rate up to 100 and 85%.

**Conclusions:**

Fatigability is an important additional dimension of physical impairments across the severity spectrum in children and adults with SMA. The EST’s are reliable and valid to document fatigability of walking, proximal- and distal arm function in SMA and thus are promising outcome measures for use in clinical trials.

## Background

Hereditary proximal Spinal Muscular Atrophy (SMA) is a severe neuromuscular disorder with predominantly infantile or childhood onset and is caused by deficiency of the survival motor neuron (*SMN*) protein due to loss of function of the *SMN1* gene [1]. SMA is characterised by progressive loss of muscle strength and motor function with a large clinical variety ranging from severe hypotonia in the first months of life (type 1), stalled gross motor development but the ability to sit without support (type 2), difficulties with or the loss of ambulation later in life (type 3) to relatively mild impairments in adulthood (type 4) [2–5]. Fatigability, defined as the inability to sustain repetitive physical activities, is increasingly being recognized as an important additional dimension of physical impairments and a target for therapeutic interventions [6–9]. Research into the effect of both SMN-augmenting treatment strategies and pharmacological compounds specifically targeting skeletal muscle on fatigability is hampered by the lack of sensitive and clinically relevant outcome measures for the assessment of fatigability [10–13]. Therefore, we recently established content validity and feasibility of the Endurance Shuttle Tests [7, 14, 15]. The *primary* objective of this study was to determine construct validity and reliability of the Endurance Shuttle - Walk Test, − Box and Block Test and – Nine Hole Peg Test as outcome measures for fatigability of walking, proximal- and distal arm function in SMA types 2–4. The *second* objective was to compile and evaluate the Endurance Shuttle Test Combined Score to increase sensitivity and provide one single outcome measure for a broad range of phenotypes.

## Methods

### Subjects

Patients with SMA type 2, 3a, 3b and 4 were recruited from the Dutch national SMA registry (ww.treatnmd.eu/patient registries) [2, 16]. To minimize selection bias, all eligible patients from a total of more than 300 enrolled in this register were invited to participate. All patients had a confirmed homozygous deletion of the *SMN1* gene or a heterozygous *SMN1* deletion in combination with a disabling point mutation on the second *SMN1* allele. Disease controls with another (genetically) confirmed neuromuscular disease were recruited from the paediatric neuromuscular outpatient clinic at the University Medical Center Utrecht and from Rijndam Rehabilitation Center in Rotterdam, the Netherlands. Healthy controls were recruited from the HU University of Applied Sciences, the University Medical Center Utrecht and through the subject’s social network of family, friends and schoolmates. Inclusion criteria were an age between 8 and 60 years and the ability to follow test instructions. Subjects were excluded if they had a history of Myasthenia Gravis or another neuromuscular disorder known to cause fatigability or affect neuromuscular junction function, if they used drugs that change neuromuscular transmission, or if they had other medical problems that could interfere with the outcomes of the testing.

### Study design

The study consisted of three visits (V1,V2,V3) within approximately 6 weeks (Table 1). At V1 we documented baseline characteristics and subjects practiced the endurance tests during 1 min to reduce the learning effect on test-retest reliability. At V2 and V3, subjects performed respectively test 1 (test) and test 2 (retest) at home or at the exercise laboratory in our hospital (both under supervision), depending the subjects preference. There was at least 1 week resting period between V2 and V3.

**Table 1:** Study design

	V1	V2	V3
Baseline			
Demographics	X		
Medical history	X		
Muscle strength	X		
Endurance Shuttle Tests			
Practice test	X		
Endurance test 1 (test)		X	
Endurance test 2 (re-test)			X

### Muscle strength

We assessed muscle strength of 22 muscle groups on both sides using a slightly modified Medical Research Council (MRC) score (i.e. no distinction between MRC 0 and 1; in both cases we used a score of 1) and calculated the MRC sum score (Range: 44–220) [2]. We calculated a sub score for the upper limb strength using 11 muscle groups of the upper limb on both sides (22–110).

### Endurance shuttle tests

The Endurance Shuttle - Nine Hole Peg Test (ESNHPT), − Box and Block Test (ESBBT) and - Walk Test (ESWT) were performed according to standardized procedures as previously described [7]. In short, we instructed subjects to repeatedly place and return 9 pegs in 9 holes, move 10 blocks over a partition or walk 10 m at 75% of their previously determined, individualized maximum speed. The individual rounds were paced by auditory signals. The test was ended when the subject was not able to keep up the pre-set pace during two consecutive shuttles or when the maximal duration of 20 min was reached (test completion). Subjects performed all tests they were physically capable of in a predetermined order starting with the ESNHPT followed by the ESBBT and the ESWT. Subjects recovered between tests for at least 30 min. Fourteen out of 25 (56%) HC performed tests for the duration of 10 (rather than 20) minutes. This test duration was chosen for the initial protocol but was later changed into 20 min to optimize outcome [7]. We corrected for differences in test duration during statistical analysis. For each performed Endurance Shuttle Test (EST), we documented two outcomes ‘drop-out’ (Yes/No) and ‘time to limitation’ (Tlim) (sec). Drop-out was defined as the inability to endure the maximum duration of 20 min. We also documented test acceptability, defined as the willingness to perform the endurance test again in the future using a visual analogue scale (VAS) with a range of 0–10 [17].

### Fatigability parameters

We compared muscle strength, self-reported fatigue and motor adaptations before and directly after each EST. We determined the dominant side by documenting the hand that the subject used for writing or picking up a pen.

#### Changes in muscle strength

For change in muscle strength, we performed quantitative hand held myometry (type CT 3001, C.I.T. Technics, Groningen) according standardized procedures to measure maximal voluntary contraction (MVC) of five muscle groups of the dominant arm (shoulder abduction, elbow flexion, wrist extension, hand grip and pinch grip in subjects that performed the ESNHPT and ESBBT and of the dominant leg (hip flexion, hip abduction, knee extension, knee flexion and ankle dorsal flexion in subjects that performed the ESWT [18].

#### Self-reported fatigue

Subjects reported on general and local muscle fatigue with the OMNI scale of perceived exertion (0–10, [19].

#### Motor adaptations

We video-taped all patients during each EST to capture motor adaptations. Two assessors (BB, LH) independently compared four different aspects of performance of the first two and last two rounds of each EST: the disability to use different parts of the body together smooth and efficiently; increase in compensatory movements (i.e. movements used habitually to achieve functional motor skills when a normal movement pattern has not been established or is unavailable); increase in synkinesis (e.g. non-functional involuntary movement of muscles or limbs accompanying a voluntary movement) and decrease of the ability to move against gravity [20, 21]. ‘Motor adaption’ was assumed when at least one aspect was scored as abnormal and ‘no motor adaptation’ when all aspects were normal. The assessors resolved any disagreements through discussion.

## Statistical analysis

### Construct validity

Construct validity refers to the degree to which the scores of an instrument are consistent with predefined hypotheses regarding relationships to scores of other instruments (convergent validity) or differences among relevant groups (discriminative validity) [15].

#### Convergent validity

To determine convergent validity, we used a linear mixed model (LMM) to assess muscle strength and self-reported fatigue in SMA while accounting for within-subject clustering with a random intercept. Time (0 and 1) was added to the model as fixed effect. Subsequently, we added ‘drop-out’ and the interaction between ‘time’ and ‘drop-out’ as fixed effects to determine the effect of drop-out on muscle strength and self-reported fatigue. The association between drop-out and motor adaptations was studied with Pearsons Chi Square and Fisher’s exact test. We hypothesized that subjects with SMA would demonstrate a lower muscle strength, higher self-reported fatigue and more motor adaptations directly after the EST compared to before.

#### Discriminative validity

We used the log-rank test to study whether the ESWT and ESBBT could discriminate between SMA and HC and the ESNHPT between SMA, HC and DC. Event probabilities were estimated using Kaplan Meyer estimates. Group differences in age (between SMA, HC and DC) and muscle strength (between SMA and DC) were tested with Mann-Whitney U test. We hypothesized that patients with SMA would demonstrate increased drop-out rates and shorter endurance time compared to HC and DC.

### Reliability

For test-retest reliability, we calculated the two-way mixed intra-class correlation coefficients (ICC), type consistency. We defined ICC’s as ‘excellent’ if the lower bound of the 95% CI > 0.80, ‘high’ if it ranged between 0.7–0.8, and ‘moderate’ if it ranged between 0.5–0.7 [22]. For agreement between test completion of test 1 and test 2, we calculated Cohen’s kappa considering a kappa of 40–60% as moderate, 60–80% as substantial and > 80% as excellent agreement [22]. Due to repeated measurements of the time-to-event outcome (i.e. trial 1 and 2), we used a linear mixed Cox model with a Gaussian distribution to account for intra-individual clustering [23]. The linear mixed Cox model estimated the effect of retest (i.e. trial 2) on the probability of dropout and is expressed as hazard ratio. As visual illustration of test – retest effect on the dropout probability, we modeled the first test (i.e. trial 1) using a parametric Weibull model. Subsequently, we reduced the estimated Weibull hazard rate with the hazard ratio from the linear mixed Cox model.

### The endurance shuttle test combined score (ESTCS)

We compiled the ESTCS based on the scores of the separate EST’s. Patients performed, depending on their physical capability, either one (ESNHPT), two (ESNHPT, ESBBT) or all three (ESNHPT, ESBBT, ESWT) endurance tests. To compare between the most relevant endurance test of each individual, we selected the EST that corresponded with the highest level of motor function for each patient. Therefore, the ESNHPT was selected for patients with only hand- and forearm function, the ESBBT for non-ambulatory subjects who could lift their arm against gravity and the ESWT was selected for patients who could walk. For each selected EST, we documented two outcomes i.e. ‘Drop-out (Yes/No) and ‘Time to limitation’ (Tlim) (sec). The final combined outcome was adjusted for test type. We checked for normality of residuals and model assumptions. All statistical analyses were performed using SPSS for Windows (version 24.0, SPSS Inc., Chicago, Ill) and R for windows (package coxme version 2–2.10, Terry M. Therneau (2018). The sample size was not calculated prospectively because of the novelty of the endurance tests and unpredictable effect size. Sample size was determined by the number of eligible patients willing to participate.

## Results

### Subject characteristics

Sixty-one patients with SMA, 25 healthy controls and 15 disease controls completed the study (Table 2). Three participants were excluded due to perceived burden (after V1: SMA; *N* = 1), personal circumstances (after V2: HC; *N* = 1) and an injury not related to the study (after V2: DC; *N* = 1). The ESNHPT, ESBBT and ESWT were all well accepted by patients with SMA (9.0 (1.6), 8.9 (1.5), 9.1 (1.1)) and HC (9.0 (1), 9.2 (1), 9.3 (1)), respectively. The ESNHPT was moderately accepted by DC (5.8 (2.9). Respectively Both SMA and DC demonstrated a large variation in levels of muscle strength and ambulation. Patients with SMA who performed the ESNHPT were significantly older than DC (*p* = .001). General muscle strength and upper limb strength were not significantly different between SMA and DC (*p* = 0.6, *p* = 0.7).

**Table 2:** Demographics and Clinical Characteristics of Participants

	SMA (*N* = 61)				HC (*N* = 25)			PC (*N* = 15)
	ESWT (***N*** = 15)	ESBBT (***N*** = 37)	ESNHPT (***N*** = 55)	ESTCS (***N*** = 61)	ESWT (***N*** = 20)	ESBBT (***N*** = 20)	ESNHPT (***N*** = 24)	ESNHPT (***N*** = 15)
Subtype/ Diagnosis	3a: *N* = 3 3b: *N* = 11 4: *N* = 1	2: *N* = 11 3a: *N* = 10 3b: *N* = 15 4: *N* = 1	2: *N* = 30 3a: *N* = 11 3b: *N* = 14	2: *N* = 31 3a: *N* = 14 3b: *N* = 15 4: *N* = 1	NA	NA	NA	LGMD:*N* = 7 BMD:*N* = 1 DMD:*N* = 7
Gender (F:M)	4:11	17:20	34:21	34:27	12:8	14:6	15:9	3:12
Age (years)	28.4 (12.4)	28.4 (14)	28 (14.4)	28.7 (14.3)	21.8 (9.6)	21.6 (7.9)	21.1 (9.6)	14.0 (3.6)
MRC-scale (44–220)	191 (15)	161 (32)	134 (39)	138 (39)	220 (1)	220 (1)	220 (1)	144 (44)
MRC-scale upper limb (22–110)	98 (8)	86 (14)	75 (18)	76 (17)	110	110	110	79 (21)
Level of ambulation	NA: *N* = 12 CA: *N* = 2 HA: *N* = 1	NA: *N* = 12 CA: *N* = 2 HA: *N* = 3 NFA: 1 NOA: *N* = 19	NA: *N* = 9 CA: *N* = 1 HA: *N* = 2 NFA: 1 NA: *N* = 42	NA: *N* = 12 CA: *N* = 2 HA: *N* = 3 NFA: *N* = 1 NOA: *N* = 43	NA: *N* = 20	NA: *N* = 20	NA: *N* = 24	NA: *N* = 4 CA: *N* = 3 NOA: *N* = 8

*ESWT* Endurance Shuttle Walk Test, *ESBBT* Endurance Shuttle Box and Block Test, *ESNHPT* Endurance Shuttle Nine Hole Peg Test, *ESCT* Endurance Shuttle Test Combined Score, *SMA* Spinal Muscular Atrophy, *Subtype 3a* clinical symptoms < 3 yrs., *Subtype 3b* clinical symptoms > 3 yrs., *HC* Healthy Controls, *DC* Disease Controls, *MRC* Medical Research Council, *LGMD* Limb Girdle Muscular Dystrophy, *BMD* Becker Muscular Dystrophy, *DMD* Duchenne Muscular Dystrophy, *NA* Normal Ambulation, *CA* Community Ambulation, *HA* Household Ambulation, *NFU* Non Functional Ambulation, *NOA* Non Ambulation

## Construct validity and reliability

In this section we will describe outcomes of validity and reliability per separate EST and for the ESTCS.

### Endurance shuttle tests

#### ESNHPT

**Fig. 1:**
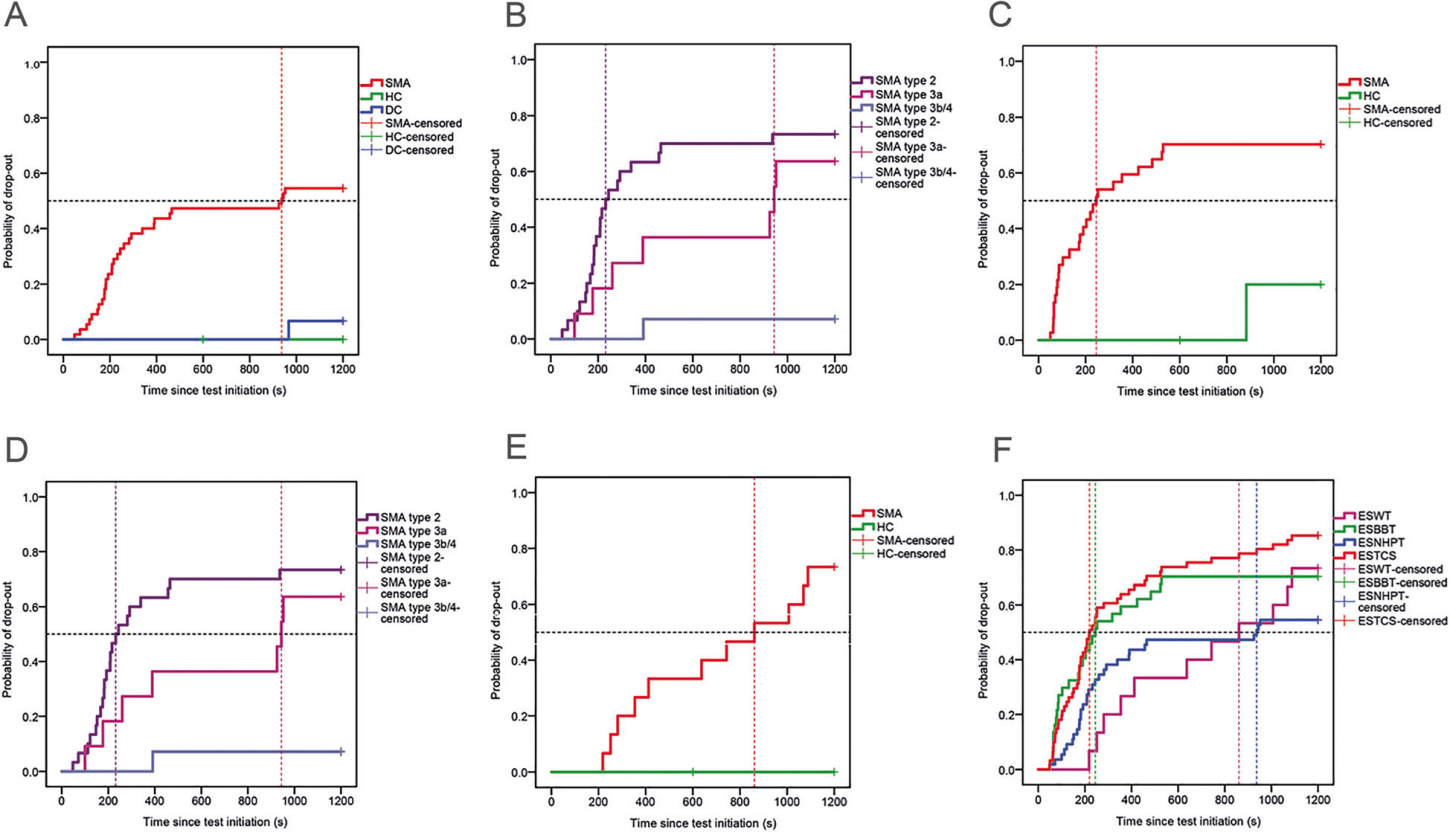
**a**-**e**. Kaplan-Meier curves of the endurance shuttle tests. Probability of drop-out since test initiation on the Endurance Shuttle Nine Hole Peg Test (ESNHPT) (**a**-**b**), Endurance Shuttle Box and Block Test (ESBBT) (**c**-**d**), Endurance Shuttle Walk Test (ESWT) (**e**) and a composite figure of all separate Endurance Shuttle Tests and the Endurance Shuttle Test Combined Score (ESTCS) (**f**). SMA: all patients with SMA; HC: Healthy Controls and DC: Disease Controls. SMA sub groups: SMA type 2, SMA type 3a and SMA type 3b and 4. Subjects that completed the Endurance Shuttle Tests are censored. The intersection of the horizontal and vertical dashed lines depict the median time to drop-out.

**Fig. 2:**
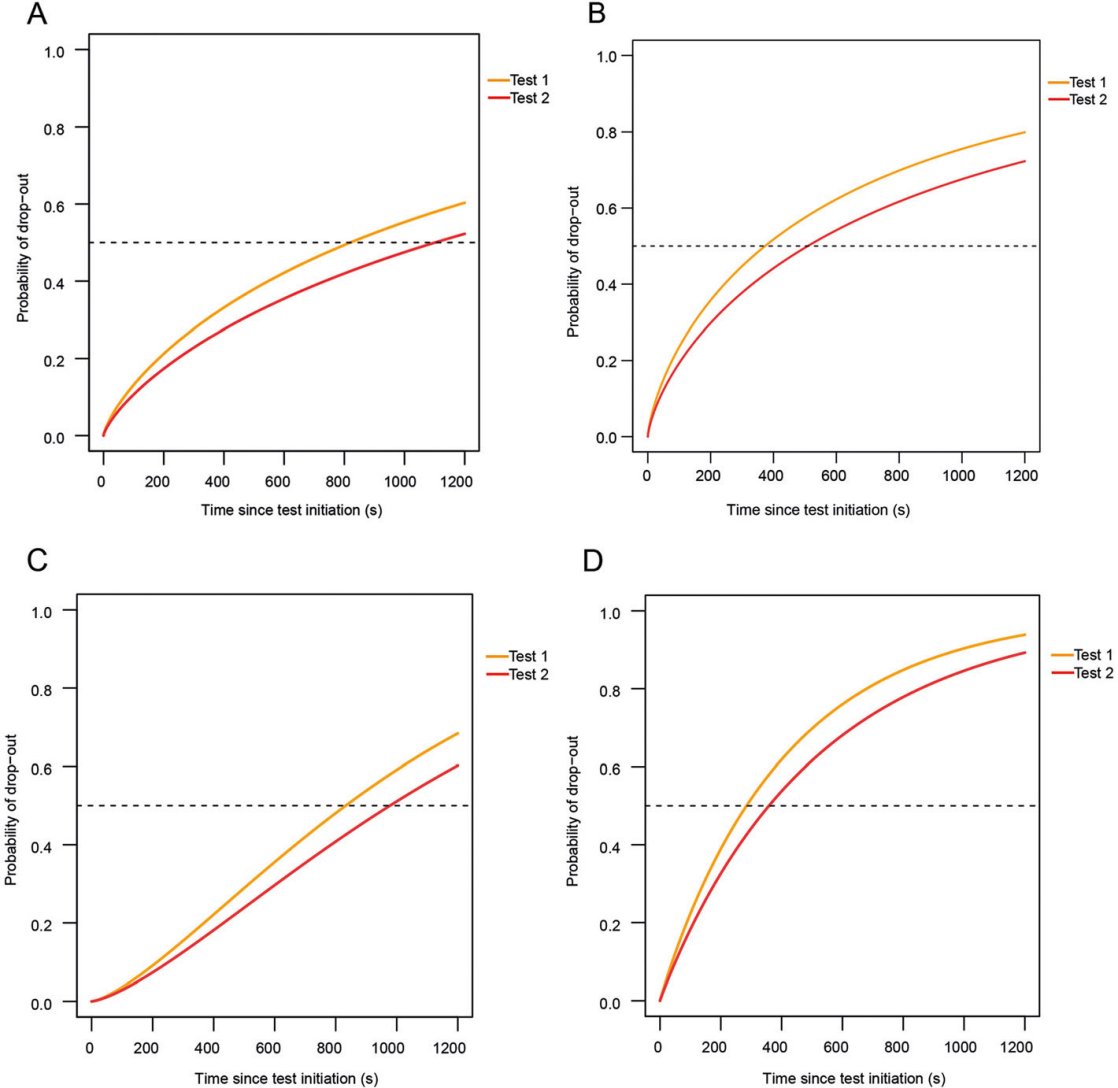
**a**-**d**. illustration of test and re-test effect on the endurance shuttle tests. Parametric Weibull curves of the Endurance Shuttle Tests. Probability of drop-out since test initiation on the Endurance Shuttle Nine Hole Peg Test (**a**), Endurance Shuttle Box and Block test (**b**), Endurance Shuttle Walk Test (**c**) and Endurance Shuttle Test Combined Score (**d**) at test 1 (orange) and test 2 (red) (**b**) in patients with SMA. The horizontal dashed line depicts the median time to drop-out.

We observed an increase in general fatigue and local muscle fatigue of the upper arm, lower arm and hand after the test in patients with SMA (Table 3). We did not find a decrease in muscle strength. Motor adaptation occurred more frequently in patients with SMA with drop-out (*p* = .000). Drop-out was significantly higher in SMA compared to HC and DC (*p* = .000) (Fig. 1a). Drop-out was different between SMA type 2, type 3a and type 3b-4 (*p* = .001) (Fig. 1b). The test-retest reliability was moderate (Table 4). Agreement on test completion between test 1 and test 2 was substantial. We observed a trend towards better performance on retest but this was not significant (Fig. 2a).

**Table 3:** Construct validity

	ESNHPT	ESBBT	ESWT
Discriminative validity	SMA – HC - DC	SMA - HC	SMA - HC
*Time to limitation* *(Mdn (s))*	SMA (*N* = 55): 937 (48–1200) HC P1 (*N* = 15): 600 HC P2 (*N* = 9): 1200 DC (*N* = 15): 1200 (967–1200)	SMA (*N* = 37): 245 (50–1200) HC P1 (*N* = 15): 600 HC P2 (*N* = 5): 1200	SMA (*N* = 15): 861 (218–1200) HC P1 (*N* = 12): 600 HC P2 (*N* = 8): 1200
*Drop-out (%)*	SMA: 54.5% HC: 0% DC: 6.7% SMA type 2: 73.3% SMA type 3a: 63.6% SMA type 3b: 7.1%	SMA: 70.3% HC: 5% SMA type 2: 100% SMA type 3a: 80% SMA type 3b-4: 44%	SMA: 73.3% HC: 0%
Convergent validity	SMA	SMA	SMA
*Muscle strength (N)*	No decrease	SA: −5.5 (2.1), *p* = .013	KF: - 8.9, *p* = .011
*Perceived fatigue (0–10)*	G: + 1.1 (0.2), *p* = .000 UA: + 2.6 (0.3), *p* = .000 LA: + 2.8 (0.4), *p* = .000 H: + 2.4 (0.4), *p* = .000	UA: + 2.6 (0.4), *p* = .000) LA: + 2.1 (0.4), *p* = .000 H: + 1.0 (0.3), *p* = .000	G: + 3.7 (0.77), *p* = .000 UL: + 4.0 (.92), *p* = .001
*Motor adaptation (Yes)*	No drop-out: 26% Drop-out: 96%	No drop-out: 33% Drop-out: 96%	No drop-out_:_ 75% Drop-out: 100%

*ESWT* Endurance Shuttle Walk Test, *ESBBT* Endurance Shuttle Box and Block Test, *ESNHPT* Endurance Shuttle Nine Hole Peg Test, *HC* Healthy Controls, *DC* Disease Controls, *P1* protocol 600 s, *P2* protocol 1200 s, *KF* Knee Flexion, *SA* Shoulder Abduction, *G* General, *UL* Upper Limb, *UA* Upper Arm, *LA* Lower Arm, *H* Hand

**Table 4:** Reliability

Test-retest reliability	ESWT (*N* = 15)	ESBBT (*N* = 37)	ESNHPT (*N* = 55)	ESTCS (*N* = 61)
Endurance time (ICC)	.91 (.77–.97)	.86 (.75–.93)	.78 (.66–.87)	.71 (.57–.81)
Test Completion (kappa)	.84 (.55–1.00)	.80 (.59–1.00)	.74 (.56–.92)	.57 (.26–.88)
Survival curves (HR)	.83 (.31–2.25), *p* = .071	.80 (.43–1.5), *p* = .49	.79 (.44–1.42), *p* = .44	.76 (.49–1.18), *p* = .22

*ESWT* Endurance Shuttle Walk Test, *ESBBT* Endurance Shuttle Box and Block Test, *ESNHPT* Endurance Shuttle Nine Hole Peg Test, *ESTCS* Endurance Shuttle Test Combined Score, *ICC* Intra Class Coefficient, *HR* Hazard Ratio.

#### ESBBT

We observed a decrease in muscle strength of shoulder abduction and an increase in muscle fatigue of the upper arm, lower arm and hand after the test in patients with SMA (Table 3). We didn’t find a significant difference between patients with and without drop-out. Motor adaptation occurred more frequently in patients with SMA with drop-out (*p* = .000). Drop-out was significantly higher in SMA compared to HC (*p* = .000) (Fig. 1c). Drop-out was different between SMA type 2, type 3a and type 3b-4 (*p* = .001) (Fig. 1d). The test-retest reliability was high (Table 4). Agreement on test completion between test 1 and test 2 was excellent. We observed a trend towards better performance on retest but this was not significant (Fig. 2b).

#### ESWT

We observed a decrease in muscle strength of knee flexion, an increase in general muscle fatigue and upper leg muscle fatigue, and an increase in motor adaptations after the test in patients with SMA (Table 3). We didn’t find a significant difference between patients with and without drop-out. Drop-out was significantly higher in SMA compared to HC (*p* = .000) (Fig. 1e). The test-retest reliability was high and agreement on test completion between test-retest was excellent (Table 4). We observed a trend towards better performance on retest but this was not significant (Fig. 2c).

### Endurance shuttle test combined score

Drop-out (85%) was significantly higher and Time to limitation (220, 95% CI 174–266) significantly lower on the ESTCS compared to the separate EST’s (*p* = .002) (Fig. 1f). The test-retest reliability and agreement between test 1 and test 2 were moderate (Table 4). We observed a trend towards better performance on retest but this was not significant (Fig. 2d).

## Discussion

The primary objective of this study was to determine construct validity and reliability of the EST’s in patients with SMA. Results of our study indicate good convergent validity of EST’s to assess fatigability and good discriminative validity between patients with SMA, HC and DC. Even with similar muscle strength, higher frequency of drop-out and shorter endurance time in patients with SMA were present compared to disease controls. These results indicate that fatigability is an important dimension of physical impairment in SMA separate from muscle strength.

The high prevalence of fatigability we report in both mildly and severe affected patients with SMA is consistent with recent studies that reported increased fatigability in ambulatory patients with SMA type 3 using the 6-min walk test (6MWT) and in type 2 patients with the repetitive Nine Hole Peg Test (r9HPT) [24, 25]. The 6MWT and the r9HPT however, do not cover the large severity spectrum of SMA and use different methodologies which make them difficult to compare. Therefore, we developed a set of endurance shuttle tests based on the same construct using the same methodology in patients with mild, moderate and severe motor impairments [7]. The ESNHPT showed increased sensitivity of approximately 64% to capture fatigability during fine motor tasks in patients with SMA type 3a compared to 36% using the r9HPT [25]. The ESBBT is the first validated and sensitive fatigability test for proximal arm function in SMA and may be complementary to outcome measures that focus on arm motor function such as the Revised Upper Limb Measure (RULM), by adding the dimension of endurance [26]. Few studies have addressed the prevalence of fatigability and the variability in endurance capacity between ambulatory patients [24, 27]. Our results show that most ambulatory patients do show fatigability during walking, but that the moment at which that occurs is highly variable. The fact that respectively over 80% of the patients with SMA were able to walk for more than 6 min at a constant walking speed during the ESWT, does suggest that the currently used 6MWT might not be sensitive to capture fatigability in patients with moderately limited ambulatory capacity. The ESWT could be a good alternative to capture change in endurance in ambulatory patients. The reliability of the EST’s was good (ICC’s .78–.91) and similar to the r9HPT and 6MWT (ICC’s .71–.99) [25, 28]. Reliability of the ESNHPT was slightly lower than the ESBBT and ESWT which was explained primarily by a learning effect we observed in some videos. We did not detect a learning effect in a previous study on the value of the r9HPT to document fatigability in SMA, so we anticipated that a practice session of 1 min would be sufficient to correct for motor learning [25]. Based on the findings in this study, a complete practice test of the entire duration of 20 min should be applied in the future. Ideally, outcome measures can be used across the severity spectrum of SMA without large floor- and ceiling effects. These and previously published data of motor function and endurance suggest that current performance measures are not sensitive to capture possible changes at the extreme ends of the spectrum of physical abilities [25, 29]. A commonly used method to counteract this problem in functional scales, adding items to both ends of the hierarchical scale, is not applicable to exercise testing [26, 30, 31]. The second objective of this study was to develop a combined score that would allow comparison of patients with varying severity on their individual most relevant endurance test, thereby increasing sensitivity and circumventing subgroup analysis with less statistical power. The ESTCS increased sensitivity to detect fatigability and increased sample size compared to the ESNHPT (+ 31%, *N* = + 6), the ESBBT (+ 15%, *N* = + 24) and the ESWT (+ 12%, *N* = + 46). At the same time, test-retest reliability of the ESTCS was slightly lower compared to the reliability of the individual EST’s. This implies that in the choice between a separate EST and the ESTCS, the size and heterogeneity of the study sample and the degree of reliability and sensitivity that are necessary to demonstrate trial efficacy have to be taken in account. An important strength of this study was the application of survival analysis to quantify fatigability in SMA which gave us the opportunity to include patients with severe fatigability that could only sustain the specific endurance test for a short amount of time. The alternative method that looks at change over time such as the 6MWT or repetitions such as the r9HPT might underestimate fatigability because patients that drop out early are often not included in the analysis. The use of hazard ratios is an innovative approach to test reliability and can be used to determine efficacy of clinical trials by calculating the difference with the hazard ratio of the treatment- versus placebo group. Longitudinal natural history studies and data from clinical trials are now required to determine whether the EST’s are sensitive to detect clinically meaningful changes over time. We were not able to determine discriminative validity of the ESWT and the ESBBT between SMA and DC since few patients with Muscular Dystrophy we included were able to walk or lift their arms against gravity. Disease controls are generally hard to recruit and difficult to match with SMA on the severity and distribution of muscle weakness. Despite the limited number of DC’s, we made a first step to explore differences in fatigability response between subjects with SMA and other neuromuscular diseases. The lower endurance time in patients with SMA compared to DC is in line with previous results using the repetitive nine hole peg test [25]. The available data suggest that the dramatic deterioration in muscle performance that we observed in many subjects with SMA, is not present to the same extent in disease controls even with similar muscle strength, but this needs further confirmation.

## Conclusion

We show that the Endurance Shuttle Tests are reliable and valid to assess fatigability in patients with SMA across the spectrum of disease severity. This makes them promising outcome measures for application in standard care and clinical trials in patients with SMA.

## Data Availability

The datasets used and/or analysed during the current study are available from the corresponding author on reasonable request.
